# Long-Term Course of Hypothyroidism Detected through Neonatal TSH Screening in a Population-Based Cohort of Very Preterm Infants Born at Less than 32 Weeks of Gestation

**DOI:** 10.3390/ijns7040065

**Published:** 2021-10-13

**Authors:** Birgit Odenwald, Aline Fischer, Wulf Röschinger, Bernhard Liebl, Heinrich Schmidt, Uta Nennstiel

**Affiliations:** 1 Newborn Screening Centre/State Institute of Health, Bavarian Health and Food Safety Authority, 85764 Oberschleissheim, Germany; Bernhard.Liebl@lgl.bayern.de; 2 Paediatric and Youth Medicine Clinic, Klinikum Dritter Orden, 80638 Munich, Germany; aline.fischer@dritter-orden.de; 3 Newborn Screening Unit, Becker and Colleagues Laboratory, 81737 Munich, Germany; W.Roeschinger@labor-becker.de; 4 Paediatric Endocrinology, Dr. von Hauner Children’s Hospital, Ludwig-Maximilians-University, 80337 Munich, Germany; Heinrich.Schmidt@med.uni-muenchen.de

**Keywords:** neonatal screening, thyroid-stimulation hormone, congenital hypothyroidism, preterm infant, follow-up, re-evaluation

## Abstract

After several decades of successful newborn screening (NBS) for congenital hypothyroidism, the optimal hypothyroidism NBS algorithm for very preterm infants is still controversial. Due to concerns about an elevated risk of a false-negative initial thyroid-stimulation hormone (TSH) screening, repeat NBS has been implemented for this group. While transient hypothyroidism is known to be more frequent among very preterm infants, the prevalence of permanent hypothyroidism is generally assumed to be the same as in more mature newborns. This study analyses screening and long-term follow-up data from the population-based cohort of 51 infants born from 1999–2017 at less than 32 weeks of gestation and diagnosed with hypothyroidism after NBS in the German Federal State of Bavaria (total number of infants screened 2,107,864). Severe permanent hypothyroidism was always detected at initial TSH screening unless there was a known confounding factor. Cases detected by repeat screening after a negative initial screen most frequently proved to be transient, less frequently mild permanent, or a definitive diagnosis was not possible because of inadequate re-evaluation of the thyroid axis. The prevalence of both permanent and transient hypothyroidism was elevated compared to a cohort of children from the same region born at a higher gestational age. The results seem to support the need for the repeated NBS of very preterm infants. However, as the recommendation to treat mild hypothyroidism is not based on high quality evidence, important issues for future research include treatment outcome studies or even a general review of whether this diagnosis meets the screening criteria. Meanwhile, involving a paediatric endocrinologist in treatment decisions is crucial for optimising the benefit of hypothyroidism screening for this particularly vulnerable group.

## 1. Introduction

The newborn screening (NBS) of primary congenital hypothyroidism is widely acknowledged as a major achievement and has been established in many programmes worldwide since its development in the 1970s. Left untreated or treated late, hypothyroidism is a cause of irreversible neurodevelopmental damage, growth retardation, and metabolic complications. Early detection and treatment of hypothyroidism by NBS has been proven to prevent adverse outcomes successfully [1,2].

The detection rate of primary congenital hypothyroidism in NBS programmes lies between 3 and 5 per 10,000 births. The most common screening strategy is the detection of elevated levels of pituitary thyroid-stimulating hormone (TSH) in dried blood spots during the first week of life. After a positive screening result, the European expert societies advise confirmatory serum testing of the thyroid hormone free thyroxine (FT4) and TSH, and, if hypothyroidism is confirmed, an immediate start of oral Levothyroxine (LT4) treatment, ideally within two weeks of birth [1]. In addition to severe hypothyroidism with FT4 concentrations clearly below the age-specific reference intervals, this treatment recommendation explicitly includes “mild hypothyroidism”, for neonates defined as an elevation of serum TSH above 20 mU/L with normal FT4 values [1].

While being a relatively straightforward matter for mature infants, the hypothyroidism screening process is posing some widely debated additional difficulties for very preterm infants [1,3,4]. The thyroid function patterns of very preterm infants have been shown to differ considerably from those of mature infants [5]. The terms “transient hypothyroxinaemia of prematurity” (THOP) and “delayed TSH elevation” summarise two main characteristics of the preterm thyroid situation that may interfere with NBS. Incomplete maturation of the hypothalamic–pituitary–thyroid (HPT) axis, the effects of neonatal medication, iodine deficiency or iodine excess, or serious neonatal non-thyroidal illness (NTI) have been described as possible causative factors of these phenomena [6,7,8,9]. Several follow-up studies have shown that thyroid function imbalance with delayed TSH rise among preterm infants often but not always proves transient [10,11]. The necessity to treat THOP, moderate TSH elevation with normal FT4, or mild transient hypothyroidism is a complex and controversial subject lacking clear evidence [1,4,12,13]. The prophylactic LT4 treatment of all preterms has not been shown to be beneficial [14] and several authors have emphasised the risks of overtreatment [10,15,16]. However, because of the neurodevelopmental dangers of hypothyroidism, there is a broad consensus among the majority of authors to treat all forms of hypothyroidism early and to re-evaluate thyroid function later, if appropriate—usually after the age of 2 or 3 years, when the highly thyroid-sensitive period of neurodevelopment has passed [1,3,17], under certain conditions from 6 months of age [1]. According to current recommendations for thyroid re-evaluation with discontinuation of LT4 therapy, slight TSH elevations below 10 mU/L are to be retested without immediately resuming therapy, while an off-therapy TSH rise ≥ 10 mU/L confirms primary hypothyroidism [1].

In order to avoid false-negative screening results, repeat NBS for preterm and low birth weight infants is broadly recommended and has been implemented into many NBS programmes [1,18]. However, there is no consensus about the necessity, optimal timing, and frequency of repeat NBS samples, and algorithms vary widely [19]. German NBS regulations stipulate a first screening of all neonates between 36 and 72 h of birth and a secondary screening of infants born at less than 32 weeks of gestation when they reach the equivalent of 32 weeks gestational age [20]. Several institutions perform supplementary thyroid function tests for preterm infants regularly.

An analysis of population-based German NBS data over a 5-year period indicated a 4-fold increased prevalence of hypothyroidism among infants born at less than 32 weeks of gestation, 73% of these with a delayed TSH rise [21]. Almost half of these cases were not detected by the official NBS algorithm but by supplementary repeat screening samples. Based on the screening results of 10,000,000 newborns in Germany from 2006–2019, the prevalence of congenital hypothyroidism was 3.1/10,000 overall, and 17.75/10,000 for very preterm infants born before 32 weeks of gestation [22]. As the nationwide NBS data do not include detailed background information and further follow-up data, it was unknown whether the observed high prevalence of hypothyroidism was due to an elevated frequency of transient or permanent forms, or both. For other countries, high proportions of transient hypothyroidism among preterm infants have been shown [11,23,24,25,26,27,28], but assessments of the prevalence of permanent hypothyroidism in this group are scarce and contradictory [26]. Most authors have presumed that the frequency of permanent hypothyroidism is equal among preterm and mature infants [10,29,30].

In the German Federal State of Bavaria, an ongoing regional population-based follow-up study was initiated in 1999 to evaluate neonatal screening. The present study used this database for an evaluation of the determinants and frequency of permanent and transient hypothyroidism among very preterm infants born at less than 32 weeks of gestation. In addition, prevalences were compared between this very preterm group and children born at higher gestational ages. Thus, this paper aims to contribute to the evidence base for the hypothyroidism screening of preterm infants.

## 2. Materials and Methods

This analysis was part of the population-based long-term follow-up study of children diagnosed with a target disease of NBS in the German Federal State of Bavaria. This study was approved by the ethics commission of the Bavarian State Chamber of Physicians (registration code 04016, approval date 23 March 2004). The detailed analysis included all children with a gestational age below 32 weeks who were born and screened in Bavaria from 1999 to 2017 and diagnosed with hypothyroidism in need of treatment during the neonatal screening process, excepting children with identified genetic syndromes or severe malformations. The analysis was aimed to distinguish between permanent and transient hypothyroidism for each child included.

Screening was performed in accordance with German guidelines for the prevention of disease in children [20]: Blood is sampled at 36 to 72 h after birth, and retesting is advised for samples taken under the influence of corticosteroid or catecholamine administration, or blood transfusion, or in infants born <32 weeks of gestation. Repeat NBS at the equivalent of 32 weeks gestational age has been advocated by paediatric societies for the entire observational period of this study and was incorporated into the official regulations in 2005. TSH was measured from dried blood filter cards by immunological assays (AutoDELFIA^®^) in the two specialised laboratories licensed for neonatal screening in Bavaria. TSH screening cut-off values were <20 mU/L for the first days of life, <15 mU/L for days 2–5 (laboratory 1) or 4–5 (laboratory 2), and <10 mU/L from day 6.

Tracking of abnormal values and the long-term follow-up study were carried out at the Bavarian screening centre, which for both screening laboratories is located at the Bavarian Health and Food Safety Authority. Follow-up information was obtained via medical reports and parental questionnaires or, in some cases, telephone inquiries with families or paediatricians. Parents’ informed consent was obtained separately for screening, tracking, follow-up by questionnaires, and follow-up by medical records. With some differences in methodology and a shorter follow-up period, data on part of the present cohort have already been reported in a German-language dissertation [31].

Diagnostic criteria were assessed in accordance with the European Consensus Guidelines on Screening, Diagnosis, and Management of Congenital Hypothyroidism published by the European Society for Paediatric Endocrinology (ESPE) in 2014 [3] and updated in 2020 [1].

Diagnostic criteria for the inclusion into the analysis were the biochemical conditions which, according to these guidelines, clearly indicate the decision to initiate LT4 treatment in case of neonatal screening TSH elevations: Serum TSH ≥ 20 mU/L, or TSH elevated, but lower than 20 mU/L and FT4 below cut-off. Serum FT4 lower cut-off was set 10 pmol/L if there was no laboratory-specific FT4 normative value range given. Serum FT4 was rated very low if <5 pmol/L and low if 5–<10 pmol/L.

The final diagnosis of permanent hypothyroidism required at least one of the following conditions:

1. Conclusive confirmation of thyroid dysgenesis, thyroid ectopy, or athyreosis, on imaging.
2. Confirmation of severe dyshormonogenesis by molecular genetic testing.
3. Positive result of off-treatment re-evaluation of the thyroid axis, or positive result of LT4 dose decreasing trial (= increase in TSH concentration to ≥10 mU/L after 4 weeks off-treatment or after 2 to 3 weeks of LT4 dose decreased by 30%).
4. Rise of venous TSH concentration under ongoing LT4 treatment after the first year of life, in combination with repeated increases of LT4 dosage, age and weight appropriate, over years with regular controls of thyroid function.

In addition, the final diagnosis “permanent” was further differentiated:

- Severe permanent hypothyroidism when there was evidence of a significant morphologic abnormality of the thyroid gland or evidence of a decreased FT4 on re-evaluation of thyroid function.
- Mild permanent hypothyroidism when there was no evidence of a morphologic abnormality of the thyroid gland and a TSH increase to above 10 mU/L with FT4 in the normal range was detected on re-evaluation.

The final diagnosis of transient hypothyroidism or TSH elevation required a steady euthyroid state (TSH and FT4 normal) after discontinuation of LT4 therapy.

The final diagnosis was classified inconclusive if none of the “permanent” criteria was fulfilled but LT4 therapy was continued, and re-evaluation of the thyroid axis was inadequate or not carried out until the 4th year of life or later. The classification unknown was used if sufficient information for a final diagnosis was not available because of loss to follow-up.

For prevalence comparison with children born at a higher gestational age, the same inclusion and diagnostic criteria were applied to the corresponding subgroup of the Bavarian population-based long-term study. Follow-up of all children covered the birth years from 1999 to 2013. To increase the number of very preterm cases, follow-up was extended up to the birth year of 2017 for infants born <32 weeks. Therefore, the calculations of prevalence refer to birth years 1999–2013 for births ≥32 weeks of gestation, and to 1999–2017 for births <32 weeks. With a screening rate of 98.6%, the screening database included all children born, screened, and registered in Bavaria. Reference total birth numbers were 1,615,592 births ≥32 weeks of gestation (1999–2013) and 23,171 births <32 weeks of gestation (1999–2017). Prevalences were calculated per 10,000 births of each gestational age group.

Screening and tracking data were documented in Access^®^ (1999–2012) and Oracle (since 2013) databases at the screening centre. All relevant data were entered retrospectively into a separate pseudonymised Access^®^ database and analysed with IBM^®^ SPSS^®^ Statistics for Windows, Version 25.0. (IBM Corp., Armonk, NY, USA) software, using non-parametric tests at a level of significance of *p* < 0.05, where appropriate.

## 3. Results

Among all the infants born, screened, and registered in Bavaria from 1999 to 2017 (*N* = 2,107,864; screening rate 98.6%), a total of 51 infants born preterm at a gestational age of less than 32 weeks had a diagnosis of hypothyroidism treated with LT4 after neonatal screening and were included in this analysis. Prevalence of treated hypothyroidism thus was 22:10,000 in this subgroup of preterms (number of births <32 weeks of gestation *N* = 23,171).

**Figure 1:**
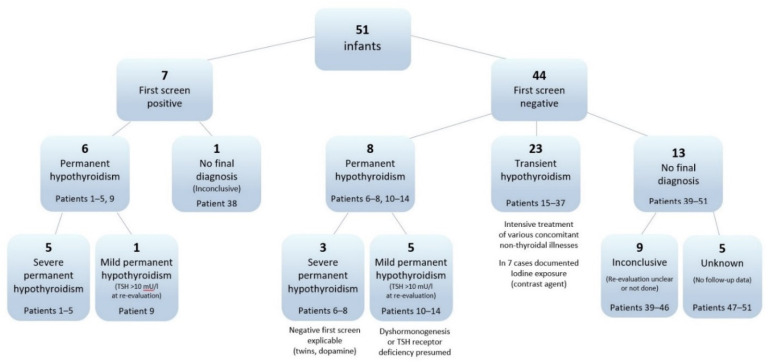
Overview of first screen results and final diagnoses.

For five children follow-up information was unavailable. One of these children died at the age of 7 months, two families declined follow-up, and two families were lost to follow-up. Of the 46 children with follow-up information, for 37 children a definite diagnosis of permanent or transient hypothyroidism could be established, while for 9 children evidence was inconclusive (Figure 1).

Table 1 shows the main characteristics of the study population, Table 2 shows the individual characteristics grouped by final diagnoses.

### 3.1. Permanent Hypothyroidism

Permanent hypothyroidism was confirmed for 14 children (27.5%), namely, 6 children with a positive result of initial screening (Table 2, patients 1–5 and 9) and an additional 8 children with a negative result of initial screening, detected after a positive screening control (patients 6–8 and 10–14). On further differentiation, eight cases were classified as severe permanent hypothyroidism (patients 1–8) and six cases as mild permanent hypothyroidism (patients 9–-14). Among the eight children with confirmed severe hypothyroidism, five had a positive initial screening (patients 1–5) and three had an explicably negative initial screening, the causative factors being feto-fetal transfusion by an identical euthyroid twin (patients 6 and 7) or high-dose dopamine treatment (patient 8). In four initially negative cases with the final classification of mild hypothyroidism, there were indications to suspect a hereditary form of hypothyroidism (patients 10, 12–14).

### 3.2. Transient Hypothyroidism

Transient hypothyroidism was confirmed for 23 children (45.1%), all of whom had a negative initial screening result and critical concomitant illnesses with various medications in the neonatal intensive care unit. Maternal thyrostatic treatment or positive maternal antibodies were not mentioned in any of these patients’ medical reports. An exposure to an iodine contrast agent preceding the TSH rise was documented for seven of these infants. Decreased FT4 levels before initiation of therapy were found in 12 of these cases (patients 15–26). Treatment was terminated during the first year of life in eight cases (34.8%), during the second year of life in five cases (21.7%), in the third year of life in six cases (26.1%), and at age three years or older in four cases (17.3%).

### 3.3. Comparison of Permanent and Transient Groups

**Figure 2:**
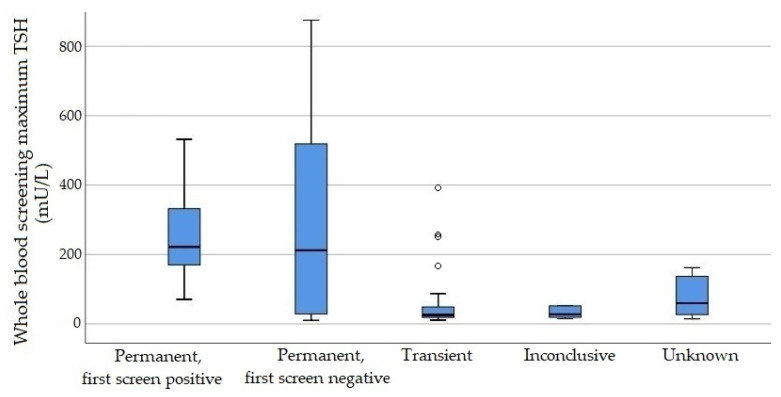
Maximum TSH screening values (whole blood, mU/L). Boxes represent median and interquartile range (IQR). Whiskers range between the minimum and maximum values, excluding outliers >1.5 IQR from the box which are represented by small circles.

**Figure 3:**
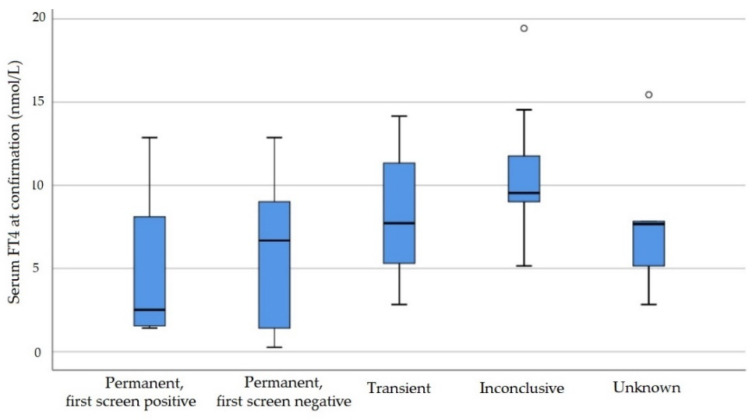
Free T4 values before initiation of treatment (serum, nmol/L). Boxes represent median and interquartile range (IQR). Whiskers range between the minimum and maximum values, excluding outliers >1.5 IQR from the box which are represented by small circles.

Maximum whole blood TSH values were significantly higher in the permanent than in the transient group (Figure 2). All infants with severe permanent hypothyroidism, 3 from 6 infants with mild permanent hypothyroidism and 4 from 23 infants with transient hypothyroidism had a whole blood TSH value above 100 mU/L at some point. Serum FT4 values before start of treatment were not available for seven infants (13.7%). Median values and distribution of FT4 measurement before start of treatment showed a tendency towards lower values in the permanent group (Figure 3), with the lowest values found in the severe permanent group (Table 2). Hypothyroxinaemia (FT4 below 10 pmol/L or TT4 below cut-off) before the start of treatment was detected most frequently in the severe permanent group (severe permanent, 6/8, 75%; mild permanent, 3/6, 50%; transient, 12/23, 52.3%). The percentage of infants with severe hypothyroxinaemia before treatment (FT4 below 5 pmol/L) was highest in the severe permanent group (5/8; 62.5%) and lower in the mild permanent and transient group (mild permanent, 1/6, 16.7%; transient, 2/23, 8.7%).

The gestational age and birth weight were significantly lower in the transient than in the permanent group, and infants with transient hypothyroidism were more often male. There was no observed difference in the frequency of infants small for gestational age (SGA) or Apgar scores between the groups (data not shown).

**Figure 4:**
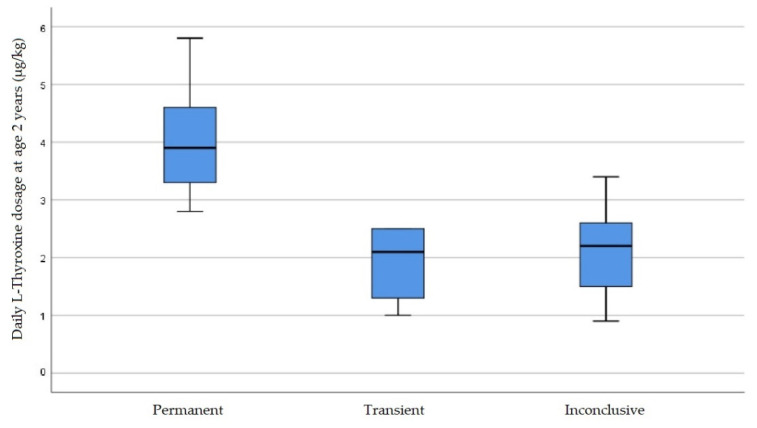
Daily dosage of Levothyroxine at the age of 2 years (µg per kg body weight). Boxes represent median and interquartile range (IQR). Whiskers range between the minimum and maximum values.

If still treated at age 2 years (permanent *n* = 14, transient *n* = 10), LT4 dosage was significantly higher in the permanent than in the transient group (Figure 4). While the highest daily dosage in the transient group was 2.5 µg/kg, the lowest dosage in the permanent group was 2.8 µg/kg (Table 2).

### 3.4. Inconclusive Cases

Establishing a conclusive diagnosis was not possible for nine children although follow-up data were available. None of these children met criteria for permanent hypothyroidism, but treatment with a steady LT4 dosage was continued beyond the age of 3 years without definite re-evaluation. A paediatric endocrinologist was not involved in the decision to continue treatment in any of these cases. An exposure to iodine contrast agent preceding the TSH rise was documented for three of these children. A family history of thyroid disorders was known for two children. Dosage at age 2 years was mostly in the range of the transient group (Figure 3).

### 3.5. Comparison of Prevalences between the Study Group and Infants Born ≥ 32 Weeks of Gestation

Overall prevalence of treated hypothyroidism or TSH elevation was nearly eight times higher among preterms born at <32 weeks of gestation (22:10,000) than among children born at higher gestational ages (2.8:10,000) (Table 3). Transient and unclear forms of hypothyroidism accounted for the most part of the observed difference. In relation to the comparison group, the relative frequency in the very preterm cohort was 2.5 for permanent hypothyroidism, and nearly 40 for transient hypothyroidism. A calculation restricted to severe hypothyroidism in the very preterm study group yielded a relative frequency of about 1.5, compared with permanent hypothyroidism overall at higher gestational ages.

## 4. Discussion

This study set out to evaluate the determinants and the epidemiology of permanent and transient congenital hypothyroidism detected through NBS in very preterm infants born at less than 32 weeks of gestation, with a focus on the impact of repeat screening samples. Main results included the detectability of severe permanent hypothyroidism at initial TSH screening in the absence of a defined confounding factor, the detection of transient and mild permanent cases through TSH screening controls, and elevated prevalences of both permanent and transient hypothyroidism in the very preterm study group.

In reviewing the literature, no research was found on population-based prevalences of permanent and transient hypothyroidism stratified by gestational age. There is, however, a relatively small body of literature that is concerned with the follow-up of preterm or low birth weight infants with hypothyroidism detected in TSH newborn screening with one or more subsequent repetitions of TSH screening. These previous studies examined the frequency of hypothyroidism within their respective cohorts without differentiating between severe and mild permanent hypothyroidism. [23,25,26,28,32,33,34]. Detailed comparability between the studies was restricted because of the diversity of selected cohorts (e.g., (very) low birth weight, or preterms below 32, 33, or 37 weeks of gestation), differing screening algorithms (timing and cut-off values) and varying case definitions. In all but one of the studies [32], hypothyroidism was classified as permanent if re-evaluation off therapy caused a TSH rise (mostly using the cut-off of 10 mU/L), regardless of an FT4 drop. For delayed TSH elevation, proportions within the previous preterm or low birth weight study cohorts ranged from 50.9 to 100% (this study, 82.4%). Hypothyroidism with delayed TSH elevation in most cases was transient, but up to 31.3% of infants with delayed TSH elevation had permanent hypothyroidism (this study, 16.6%). The lowest proportion of permanent hypothyroidism with late TSH rise (2/16; 12.5%) was reported in the only study that included FT4 values as a criterion for final diagnosis [32]. Previously reported proportions ranged from 20.5 to 50% for permanent hypothyroidism (this study, 27.5%), and from 20.8 to 84.4% for transient hypothyroidism (this study, 45.1%) among very preterm infants. Up to 15% of the cases were lost to follow-up (this study, 9.8%), and in up to 28.3% of the cases the final diagnosis remained unclear (this study, 17.6%). Taken together, consistent with the present study, previous research identified similar patterns of results, such as frequent delayed TSH elevation and frequent transient hypothyroidism, but also permanent hypothyroidism with delayed TSH elevation, among preterm or low birth weight infants.

In the present study cohort, all cases of severe permanent hypothyroidism without a known confounding factor had a high TSH value at first screening. The lowest gestational age with a positive first TSH screening was 27 weeks in this cohort, in the literature [28] and in the German nationwide NBS data (unpublished, personal communication) even 23 weeks. Thus, an immaturity of the HPT axis, widely cited as a major argument for the importance of repeat samples, did not necessarily compromise TSH screening before 32 weeks of gestation.

The observed threefold elevated prevalence of permanent hypothyroidism in the very preterm study group was unexpected [10,29] and may be connected with the high number of twins among the permanent cases, as multiple gestation pregnancies frequently lead to preterm birth and are known risk factors for congenital hypothyroidism [1,35]. In addition, because of the implementation of repeat NBS only among preterms, the detection rate of moderate dyshormonogenesis might have been expedited, e.g., the detection of DUOX2 mutations, which seem to have a potential of being undetected in initial NBS, regardless of gestational age [1,36,37,38].

In accordance with the literature, the prevalence of transient hypothyroidism was highly increased among very preterm infants born at less than 32 weeks of gestation. For the most part, one obvious reason for the observed transient hypothyroidism, like iodine exposure, could not be identified. The contribution of some immaturity or vulnerability of the HPT axis cannot be excluded, nor can iodine deficiency in an iodine-poor region with partial insufficient iodine uptake in spite of prophylactic programmes [39]. Mainly, transient hypothyroidism was likely attributed to thyroid function imbalances occurring in the context of severe neonatal morbidities and NICU treatment, the phenomenon referred to as the non-thyroidal illness (NTI) syndrome or euthyroid sick syndrome [6,7,8,40]. The observed significant male preponderance among transient hypothyroidism, as similarly reported in other publications [1], may confirm the role of NTI, reflecting the higher complication and morbidity rates among male preterm infants [41].

Despite expected tendencies of differences between the diagnostic groups, neither the extent of screening or serum TSH elevations nor FT4 values allowed a definite prognostic differentiation between permanent and transient forms, as noted in other publications [11,42]. LT4 dosage results for the age of 2 years in the study [1] cohort correlated well with the discriminative thresholds around 2–2.5 µg/kg that are found in the literature to assess the likelihood of permanence or transiency for ages 2–3 years [43,44,45].

The number of children with the final status “inconclusive”, treated with the same LT4 dose over years without the involvement of a paediatric endocrinologist, was relatively high. These cases may confirm the previously described “problem … that thyroid hormone replacement therapy is easy to start and hard to terminate” [25]. Parental concerns, paediatricians’ doubts, or ignorance regarding guideline recommendations [46] or the challenges of interpreting laboratory results after a trial off-treatment [47] may be reasons for omitted termination of therapy. A comprehensive re-evaluation of the thyroid axis in these inconclusive cases very likely would have led to a diagnosis of transient hypothyroidism, mild dyshormonogenesis [48], or receptor gene mutations [49].

While a few authors believe that repetitive TSH screening for preterm infants is unnecessary or even harmful [10,29,50], most authors and guidelines advocate repeating hypothyroidism screening in preterm and LBW infants [1,18]. The main arguments for re-screening very preterm infants, namely a general immaturity of the HPT axis or a preterm-specific problem of delayed TSH rise, are not supported by the present results but cannot be safely refuted either. From the perspective of the current guidelines [1], the results seem to confirm the necessity of at least one repeat TSH screening for very preterm infants. However, the main outcome of routine repeat screening was the detection of transient and mild cases, for which the benefit of therapy has not been proven on the basis of evidence [1,10,51], and a substantial number of children were inadequately treated longer than recommended. Thus, recommendations of repeat screenings should be complemented by measures to minimise overtreatment such as ensuring competent re-evaluation of the individual need to treat as early as possible. In addition, preterm and intensively treated children would benefit from more differentiated criteria for initiating FT4 therapy that take into account not only TSH but also FT4 and changes in thyroid function tests over time. For this, research on normative thyroid function values, adjusted both for age and gestational age would be crucial, as several authors have already stated [12,47,52,53]. Finally, the much-mentioned lack of treatment outcome studies [1,10,30,37,47], perhaps caused by ethical and methodological difficulties [10,13,51,54], appears to be a major limitation in developing screening recommendations.

This study has several limitations. The definition of the methodology was complicated by discrepancies in terminology and content among experts and in the literature. Despite the long study period and the population-based data set, the total number of cases was relatively low. The quality of medical reports and follow-up data was heterogeneous. Due to possible repeat screening gaps during the first third of the study period, the exclusion of infants with syndromes or malformations, and the number of unknown and inconclusive final diagnoses, the frequency of both transient and permanent hypothyroidism or TSH elevation among the very preterm infants may have been underestimated.

Despite its limitations, the present population-based study with a high follow-up rate confirmed previous findings and contributes to the existing knowledge on preterm hypothyroid disorders. This study included the largest number of hypothyroid infants born below 32 weeks of gestation so far and appears to be the first report on population-based stratified prevalence. Some findings of this study suggest possible courses of action on the translation of guideline recommendations into practice, such as repeat hypothyroidism screening for same-sex twins, timely initiation of thyroid re-evaluation, or inclusion of paediatric endocrine experts. Furthermore, the study clearly highlights the importance of further research on specific thyroid function normative values and controlled treatment outcome studies to enable evidence-based recommendations on hypothyroidism screening for the particularly vulnerable group of very preterm infants.

## 5. Conclusions

Most experts consider a TSH repeat screening strategy important in very preterm infants, based on reports of delayed TSH rise. In this study cohort, the majority of cases were indeed detected only through repeat screening. Still, the results could not unequivocally confirm a serious problem of delayed TSH rise in congenital hypothyroidism which would be directly associated with prematurity. Repetitive TSH screening mainly detected transient and mild cases, for which the benefit of therapy has not been proven [1,10,51], and frequently the duration of treatment was considerably longer than recommended by guidelines. Thus, it can be questioned to what extent repetitive screening strategies are compatible with the accepted screening principles [10,55] which call for a recognised therapeutic benefit [56] and state that “the overall benefits of screening should outweigh the potential harms” [57]. Evidence-based recommendations concerning the ideal hypothyroidism NBS algorithm for very preterm infants require further research. The European guideline recommendation of a one-time repeat screening at the age of two weeks most appropriately reflects the current state of research. When recommending subsequent NBS repetitions, the risks of short- and long-term overtreatment must be taken into account. At any rate, in order to avoid over-therapy and to ensure the earliest possible re-evaluation of thyroid function, for all very premature infants with hypothyroidism detected via repeat NBS, the involvement of a paediatric endocrinologist is of particular importance.

## Figures and Tables

**Table 1:** Main population characteristics and results.

Category		n	%
Gestational age (weeks)	23–25	16	31.4
	26–28	12	23.5
	29–31	23	45.1
Birth weight (BW) category	LBW (<2500 g)	6	11.8
	VLBW (<1500 g)	15	29.4
	ELBW (<1000 g)	30	58.8
	SGA	6	11.8
Sex	Male	30	58.8
	Female	21	41.2
Age at initial screening	<36 h	5	9.8
	36–72 h	41	80.4
	>72 h	5	9.8
Serum thyroid-stimulation hormone (TSH) category before start of therapy	>100 mU/L	20	39.2
	>50–100 mU/L	12	23.5
	>20–50 mU/L	16	31.4
	<20 mU/L	1	2.0
	N/A	2	3.9
Serum free thyroxine (FT4) category before start of therapy	<5 pmol/L	9	17.6
	5–<10 pmol/L	19	37.3
	10–<15 pmol/L	14	27.5
	≥15 pmol/L	2	3.9
	N/A	7	13.7
Follow-up thyroid care	Paediatric endocrinology	15	29.4
	General paediatrician	36	70.6
Final diagnostic category	Permanent hypothyroidism	14	27.5
	*severe*	*8*	*15.7*
	*mild*	*6*	*11.8*
	Transient hypothyroidism	23	45.1
	Inconclusive	9	17.6
	Unknown	5	9.8

**Table 2:** Individual patient characteristics.

Final Diagnosis After Long-Term Follow-Up	Patient Number	Sex	Birth Weight (g)	Gestational Age/Postmenstrual Age (Weeks)		Newborn Screening Results		Confirmatory Testing Results (Serum)		L-T4 Dosage at Age 2 Years (µg/kg)	Annotations
				At Birth	At Diagnosis	First Screen	Max. TSH (mU/L)	TSH (mU/L)	FT4 (pmol/L)		
**Severe permanent** **hypothyroidism**	1	f	1600	29	30	pos.	323	>100	1.4	3.8	Thyroid dysplasia. Dizygotic twin of euthyroid co-twin.
	2	m	1550	30	32	pos.	532	1365	1.7	5.8	Athyreosis.
	3	m	960	27	28	pos.	222	101	N/A	4.6	Thyroid dysplasia.
	4	f	1450	30	32	pos.	218	459	N/A	3.1	Thyroid dysplasia. Identical twin of euthyroid co-twin.
	5	m	1410	29	29	pos.	341	>100	3.4	3.8	
	6	f	1200	28	34	neg.	731	>100	<0.3	4.4	Athyreosis. Identical twin of euthyroid co-twin. Delayed re-screening in spite of tracking.
	7	f	1115	29	39	neg.	875	>100	1.5	5	Thyroid dysplasia. Identical twin of euthyroid co-twin. Born before implementation of re-screening at 32 weeks.
	8	m	460	25	28	neg.	149	280	TT4 low	3	First screen under the influence of high-dose dopamine.
**Mild permanent** **hypothyroidism**	9	f	1300	29	29	pos.	122	92	12.9	4	Family history of hypothyroidism.
	10	m	620	25	33	neg.	306	629	1.3	4.8	Dyshormonogenesis assumed.
	11	m	1730	30	40	neg.	275	>100	6.7	2.8	Negative control at age 2 weeks.
	12	f	1200	29	33	neg.	34	62	7.0	4.3	Identical twin of patient 13. Family history of hypothyroidism.
	13	f	1420	29	33	neg.	11	30	11.1	3.7	Identical twin of patient 12. Family history of hypothyroidism.
	14	m	1495	30	34	neg.	23	27	12.9	3.3	Family history of thyroid disorders
**Transient hypothyroidism**	15	m	1640	31	33	neg.	30	210	2.8	0	
	16	f	1730	31	34	neg.	87	64	3.3	0	
	17	m	870	28	32	neg.	19	19	5.1	1.7	Identical twin of euthyroid co-twin. End of therapy at 44 months.
	18	f	1060	30	32	neg.	26	77	5.1	1.2	Dizygotic twin of euthyroid co-twin. End of therapy at 27 months.
	19	m	860	27	34	neg.	254	264	5.2	2.2	End of therapy at 75 months
	20	m	670	25	34	neg.	250	33	5.4	0	Iodinated contrast agent.
	21	m	655	24	27	neg.	44	77	6.2	1.3	End of therapy at 30 months.
	22	f	1170	28	32	neg.	165	233	6.4	0	Iodinated contrast agent.
	23	m	1420	30	32	neg.	19	43	7.7	1	End of therapy at 28 months.
	24	m	740	25	29	neg.	51	78	7.7	0	Iodinated contrast agent.
	25	f	890	27	37	neg.	23	76	9.0	0	Neg. re-screening at 5 weeks.
	26	m	760	24	28	neg.	394	1004	TT4 low	2.5	End of therapy at 33 months.
	27	f	1210	29	33	neg.	13	25	10.9	0	Iodinated contrast agent.
	28	m	935	29	33	neg.	13	47	10.9	2.5	End of therapy at 43 months.
**Transient hypothyroidism**	29	m	700	24	28	neg.	13	35	11.7	0	Iodinated contrast agent.
	30	m	580	24	28	neg.	11	27	12.0	0	
	31	f	480	27	32	neg.	24	65	13.1	2.1	Iodinated contrast agent. End of therapy at 43 months.
	32	f	1095	29	37	neg.	18	51	13.1	2.5	Identical twin of euthyroid co-twin. No re-screening at 32 weeks. End of therapy at 27 months.
	33	m	410	25	27	neg.	45	44	14.2	0	
	34	m	990	30	34	neg.	28	22	14.2	0	Identical twin of euthyroid co-twin.
	35	f	620	24	28	neg.	26	97	N/A	0	Iodinated contrast agent.
	36	m	659	24	26	neg.	11	45	N/A	0	
	37	m	980	27	29	neg.	47	N/A	N/A	0	
**Inconclusive**	38	m	1780	31	32	pos.	71	N/A	11.7	3.4	Mostly extremely low TSH values under treatment, maternal autoimmune thyroiditis mentioned in medical report.
	39	m	570	24	27	neg.	52	336	5.1	0.9	Iodinated contrast agent.
	40	m	740	27	32	neg.	52	>100	5.1	1.8	
	41	f	1190	29	32	neg.	15	63	9.0	2.3	
	42	m	540	24	47	neg.	52	>100	9.0	2.1	Late treatment due to fluctuations or thyroid function tests and presumed transience.
	43	f	806	27	33	neg.	20	57	9.5	1.2	Identical twin of euthyroid co-twin. Family history of hypothyroidism.
	44	f	620	26	35	neg.	18	29	11.7	2.5	Iodinated contrast agent.
	45	m	850	31	33	neg.	32	81	14.5	2.7	Iodinated contrast agent.
	46	f	1138	31	40	neg.	24	24	19.4	2.0	Identical twin of euthyroid co-twin. Re-screening neg. at 2 weeks, pos. at 8 weeks.
**Unknown**	47	m	740	25	29	neg.	162	323	2.8	N/A	
	48	f	560	23	33	neg.	137	157	5.1	N/A	Iodinated contrast agent.
	49	f	720	25	33	neg.	60	128	7.7	N/A	Iodinated contrast agent.
	50	m	841	29	35	neg.	27	29	7.8	N/A	
	51	m	805	27	35	neg.	15	22	15.4	N/A	

f = female, m = male, neg. = negative, pos. = positive, TT4 = Total Thyroxine, N/A = Not Available.

**Table 3:** Comparison of prevalences <32 and ≥32 weeks of gestation.

	Study Group <32 Weeks of Gestation ^1^		Comparison Group ≥32 Weeks of Gestation ^2^	
	n	Per 10,000 births	n	Per 10,000 births
Permanent	14	6.0	375	2.3
Transient	23	9.9	40	0.25
Inconclusive	9	3.9	7	0.04
Unknown	5	2.2	22	0.14
Total	51	22.0	444	2.8

^1^ This analysis: birth years 1999–2017; 23,171 births <32 weeks of gestation. ^2^ Bavarian NBS follow-up study: birth years 1999–2013; 1,615,592 births ≥32 weeks of gestation.

## Data Availability

The data presented in this study are available on request from the corresponding author. The data are not publicly available due to privacy restrictions.
